# Correction: Liu, H.W.; et al. Enhanced Hsa-miR-181d/p-STAT3 and Hsa-miR-181d/p-STAT5A Ratios Mediate the Anticancer Effect of Garcinol in STAT3/5A-Addicted Glioblastoma. *Cancers* 2019, *11*, 1888

**DOI:** 10.3390/cancers12102846

**Published:** 2020-10-01

**Authors:** Heng-Wei Liu, Peter Mingjui Lee, Oluwaseun Adebayo Bamodu, Yu-Kai Su, Iat-Hang Fong, Chi-Tai Yeh, Ming-Hsien Chien, I-Hung Kan, Chien-Min Lin

**Affiliations:** 1 Graduate Institute of Clinical Medicine, College of Medicine, Taipei Medical University, Taipei City 11031, Taiwan; henryway0404@hotmail.com (H.-W.L.); yukai.su@gmail.com (Y.-K.S.); ctyeh@s.tmu.edu.tw (C.-T.Y.); mhchien1976@gmail.com (M.-H.C.); 2 Department of Neurology, School of Medicine, College of Medicine, Taipei Medical University, Taipei City 11031, Taiwan; 3 Division of Neurosurgery, Department of Surgery, Taipei Medical University-Shuang Ho Hospital, New Taipei City 23561, Taiwan; impossiblewasnothing@hotmail.com; 4 Taipei Neuroscience Institute, Taipei Medical University, Taipei 11031, Taiwan; 16625@s.tmu.edu.tw; 5 Department of Clinical Oncology, College of Medicine, California North state University, Elk Grove, California, CA 95757, USA; peter100893@gmail.com; 6 Department of Hematology and Oncology, Cancer Center, Taipei Medical University—Shuang Ho Hospital, New Taipei City 235, Taiwan; 7 Department of Medical Research and Education, Taipei Medical University—Shuang Ho Hospital, New Taipei City 235, Taiwan; 8 Department of Medical Laboratory Science and Biotechnology, Yuanpei University of Medical Technology, Hsinchu City 30015, Taiwan

The authors wish to make the following corrections to this paper [1]: 

After the publication of this work, we were notified of the mistakes in Figure 2E and Section 3.2, which have now been updated in this correction.

The original Figure 2E and legend of Figure 2E are as below:

**Figure 2:**
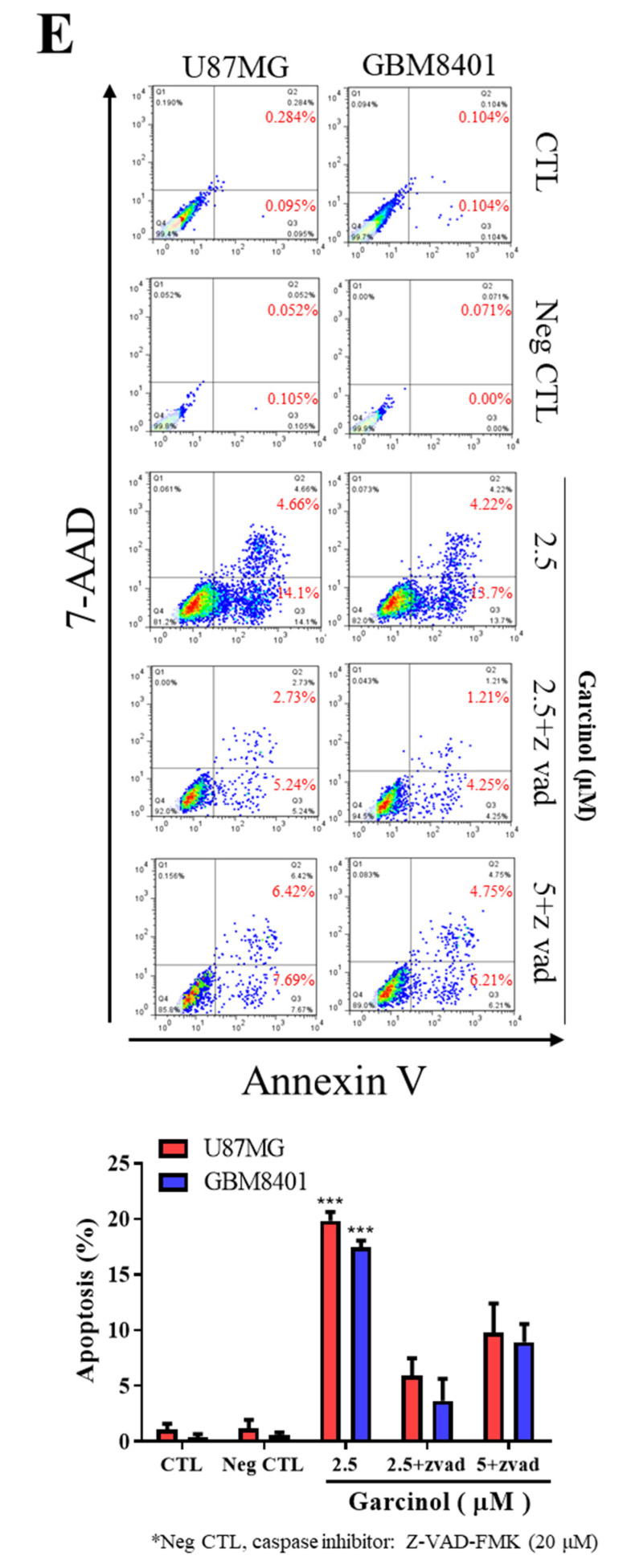
(**E**) Flow-cytometry data (upper) and graphical representation (lower) showing the effect of Garcinol, alone or in presence of Z-VAD-FMK, on U87MG or GBM8401 cells co-stained with PE-conjugated Annexin V and 7-AAD, compared with untreated control or Z-VAD-FMK-treated negative control groups. Annexin V-stained Q4 cells are early apoptotic cells, whereas Q2 cells are late apoptotic (necrotic) cells. Apoptosis (%), sum of Q4 + Q2; CTL, vehicle-treated; Neg CTL, pan-caspase inhibitor benzyloxycarbonyl-Val-Ala-Asp-fluoromethyl ketone (Z-VAD-FMK).

and should be replaced with the following: 

**Figure 2:**
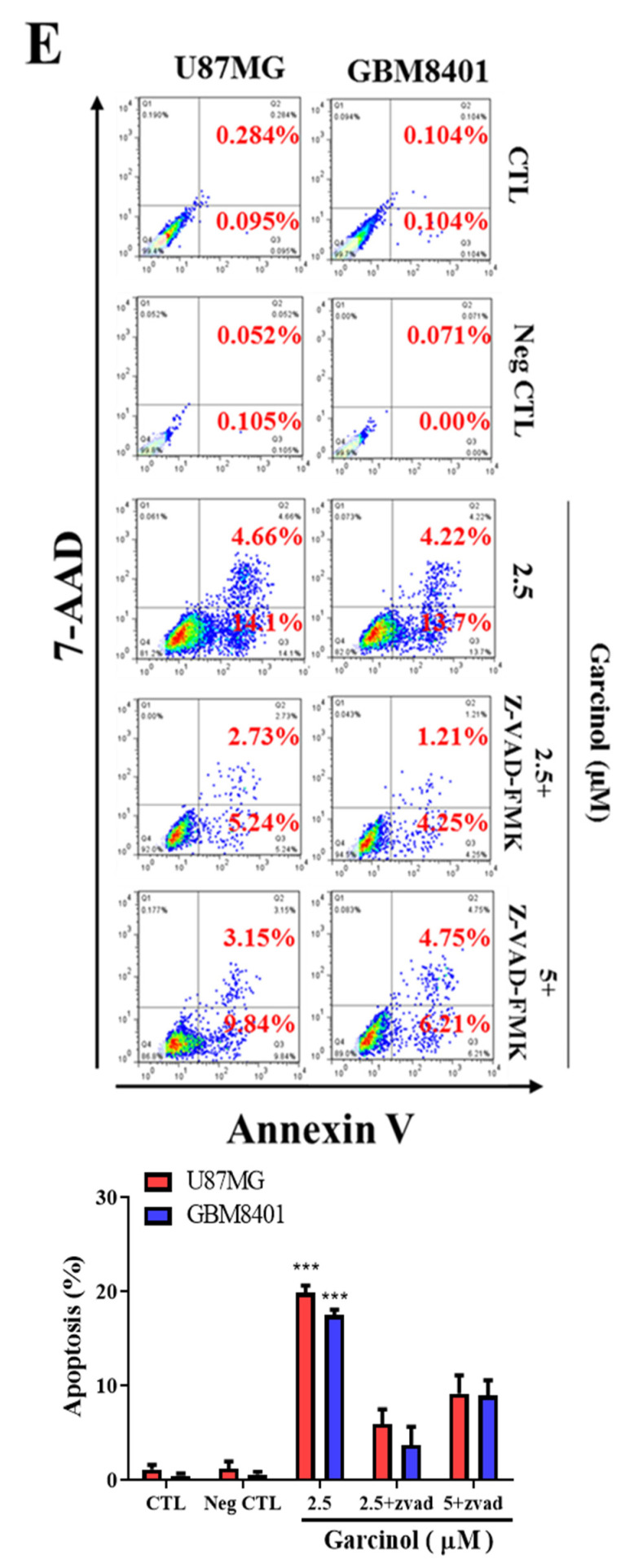
(**E**) Flow-cytometry data (upper) and graphical representation (lower) showing the effect of garcinol, alone or in presence of Z-VAD-FMK, on U87MG or GBM8401 cells co-stained with PE-conjugated annexin V and 7-AAD, compared with untreated control or Z-VAD-FMK-treated negative control groups. Annexin V-stained Q3 cells are early apoptotic cells, whereas Q2 cells are late apoptotic (necrotic) cells. Apoptosis (%), sum of Q3 + Q2; CTL, vehicle-treated; Neg CTL, pan-caspase inhibitor benzyloxycarbonyl-Val-Ala-Asp-fluoromethyl ketone (Z-VAD-FMK).

In addition, the authors reported errors in Section 3.2 when analyzing the original data in Figure 2E. Thus, the authors wish to replace the following sentence in Section 3.2: 

“…and 7.97% or 14.11% apoptosis of the U87MG cells (Figure 2E), indicating that the garcinol-induced cell death was apoptotic and caspase-dependent.”

with:

“…and 7.97% or 12.99% apoptosis of the U87MG cells (Figure 2E), indicating that the garcinol-induced cell death was apoptotic and caspase-dependent.”

The corrections made in this erratum do not affect the original conclusions. The authors would like to apologize for any inconvenience caused to the readers by these changes.
