# Supplementary material for: Enhanced Hsa-miR-181d/p-STAT3 and Hsa-miR-181d/p-STAT5A Ratios Mediate the Anticancer Effect of Garcinol in STAT3/5A-Addicted Glioblastoma
Source: Cancers (Basel). 2019 Nov 27;11(12):1888. doi: 10.3390/cancers11121888 (PMC6966688; doi:10.3390/cancers11121888)
Supplement: Supplementary file 1 [file cancers-11-01888-s001.pdf]

Supplementary Materials

Enhanced Hsa-miR-181d/p-STAT3 and Hsa-miR-181d/p-STAT5A Ratios Mediate the Anticancer Effect of Garcinol in *STAT3/5A*-Addicted Glioblastoma

Heng-Wei Liu, Peter Mingjui Lee, Oluwaseun Adebayo Bamodu, Yu-Kai Su, Iat-Hang Fong, Chi-Tai Yeh, Ming-Hsien Chien, I-Hung Kan and Chien-Min Lin

Table S1. Western blot antibodies sheet used in this study.

No.	Target	Dilution	Source	
1	Jak2	1:1000	#3230	cellsignaling
2	Stat3	1:1000	#9132	cellsignaling
3	p-Stat3	1:1000	#9145	cellsignaling
4	Stat5	1:1000	ab227687	abcam
5	p-Stat5	1:1000	ab32364	abcam
6	ERK	1:1000	#4695	cellsignaling
7	p-ERK	1:1000	#4370	cellsignaling
8	Akt	1:1000	#2920	cellsignaling
9	p-Akt	1:1000	#4060	cellsignaling
10	Bax	1:1000	#5023	cellsignaling
11	Bcl-xL	1:1000	#2764	cellsignaling
12	SOX2	1:500	#3579	cellsignaling
13	OCT4	1:500	#2840	cellsignaling
14	N-Cadherin	1:1000	#13116	cellsignaling
15	E-Cadherin	1:1000	20874-1-AP	PROTEINTECH
16	Vimentin	1:1000	#5741	cellsignaling
17	Slug	1:1000	#9585	cellsignaling
18	GAPDH	1:10000	10494-1-AP	PROTEINTECH

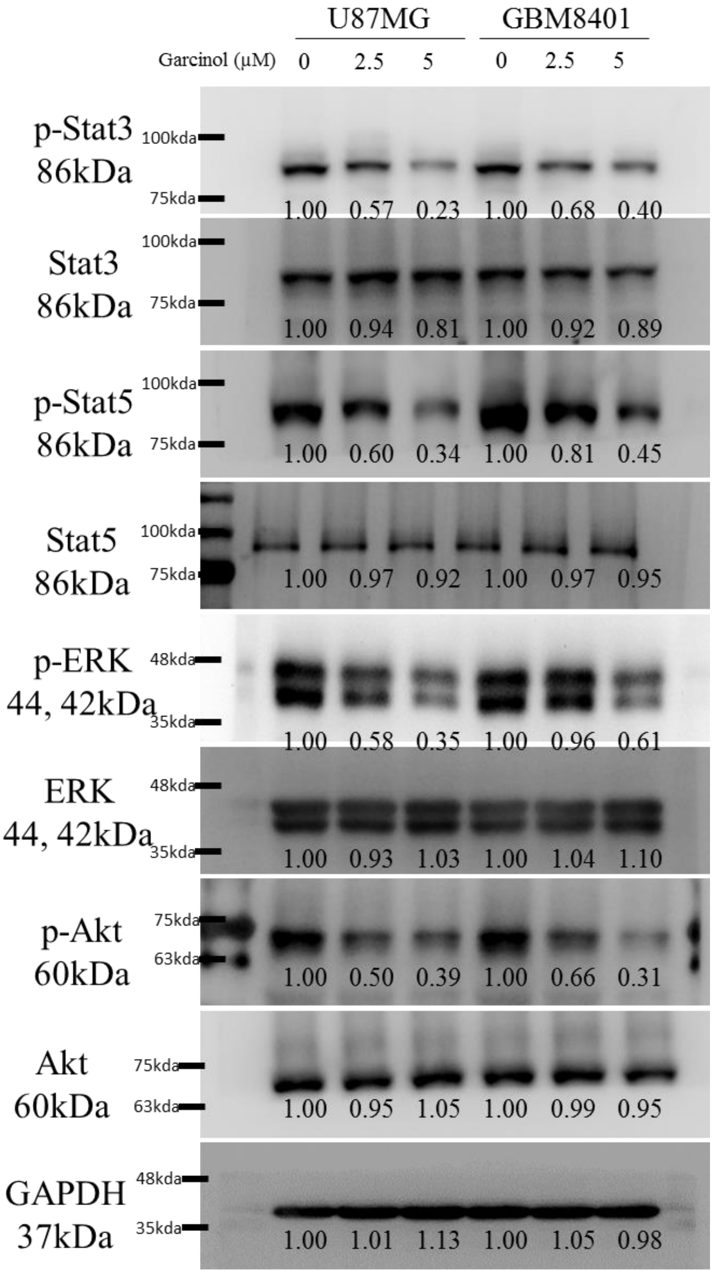

Figure S1. Full-size blots of Figure 2B.

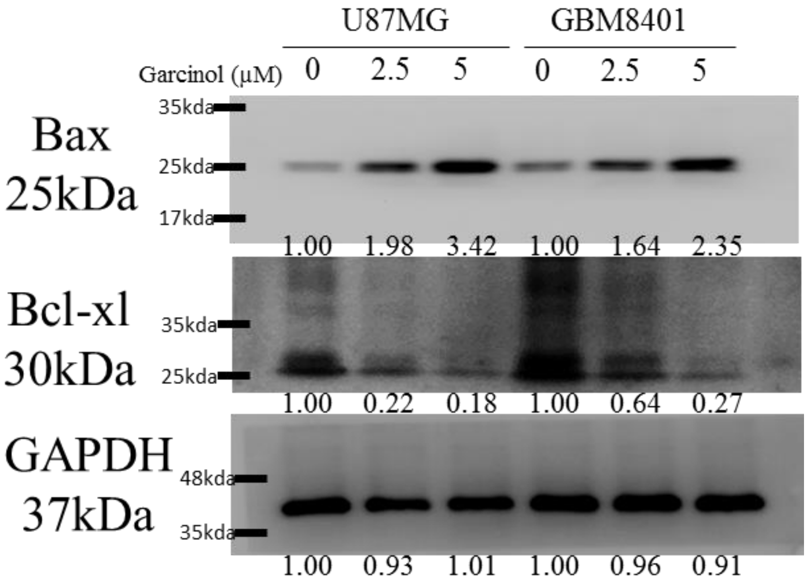

Figure S2. Full-size blots of Figure 2D.

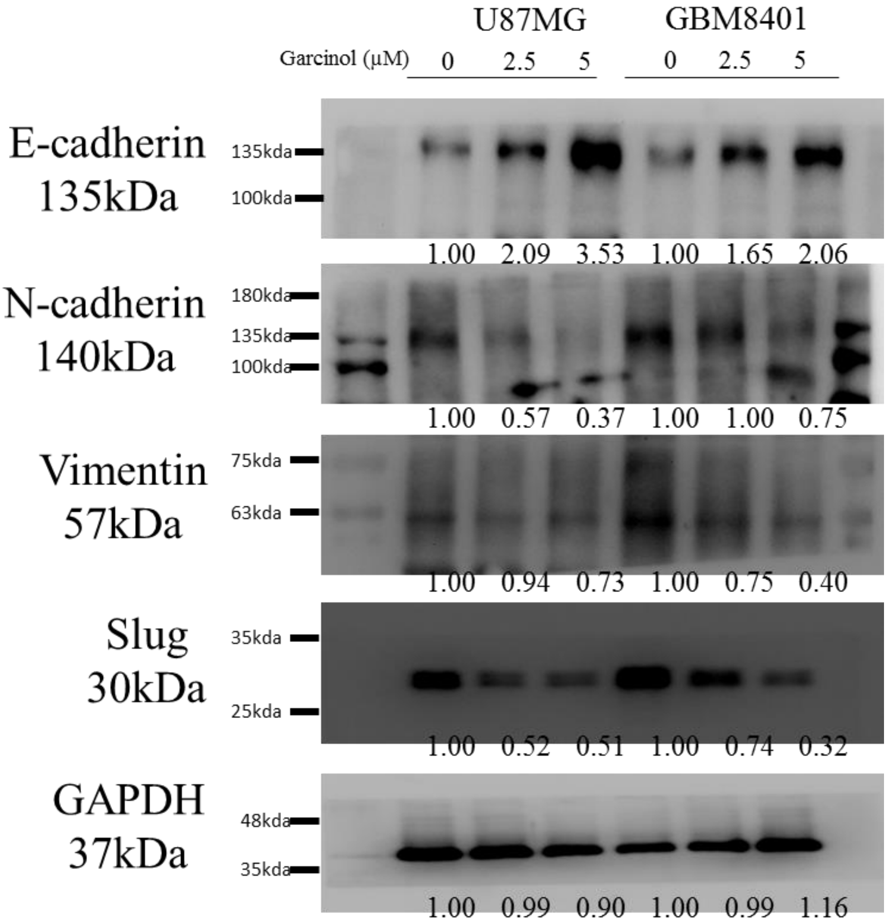

Figure S3. Full-size blots of Figure 2H.

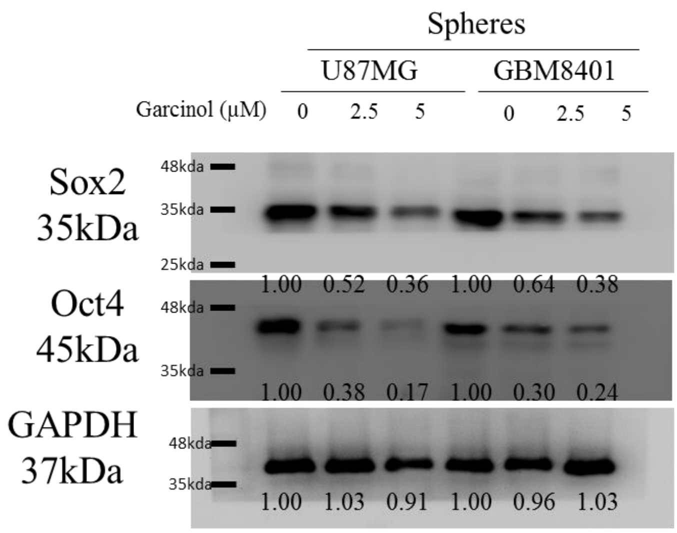

Figure S4. Full-size blots of Figure 3E.

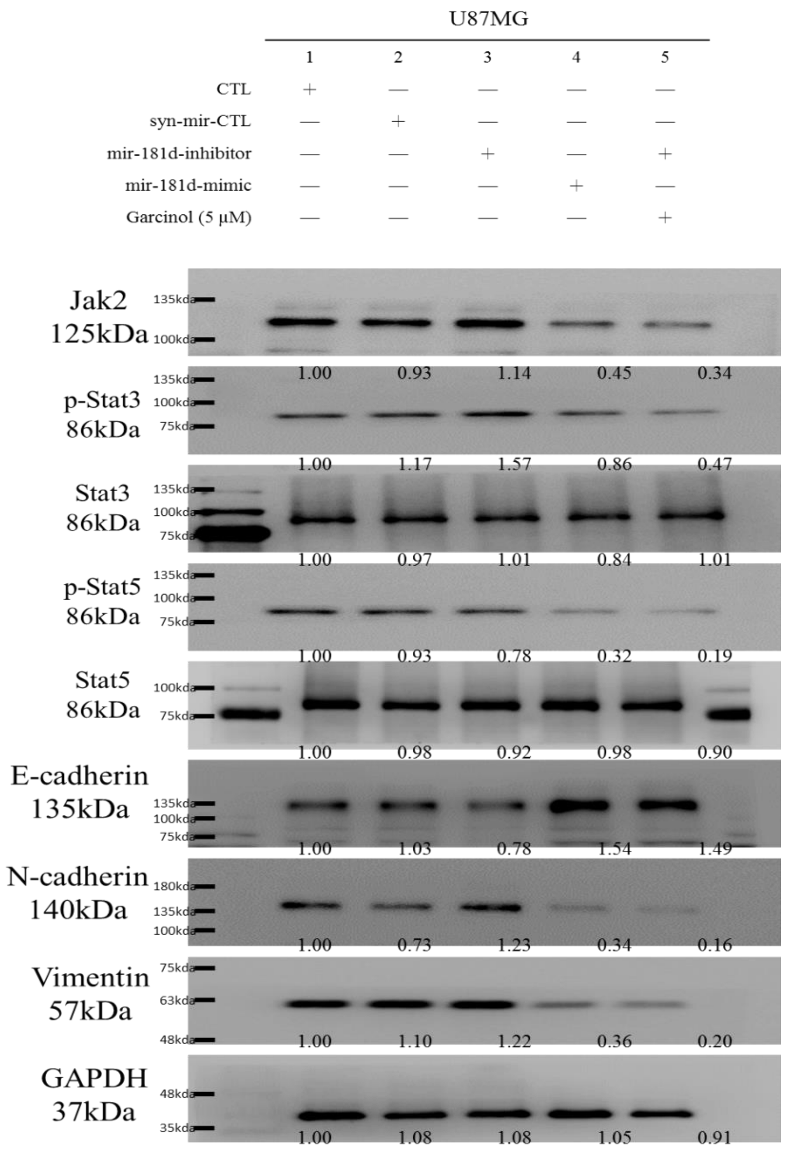

Figure S5. Full-size blots of Figure 4E.

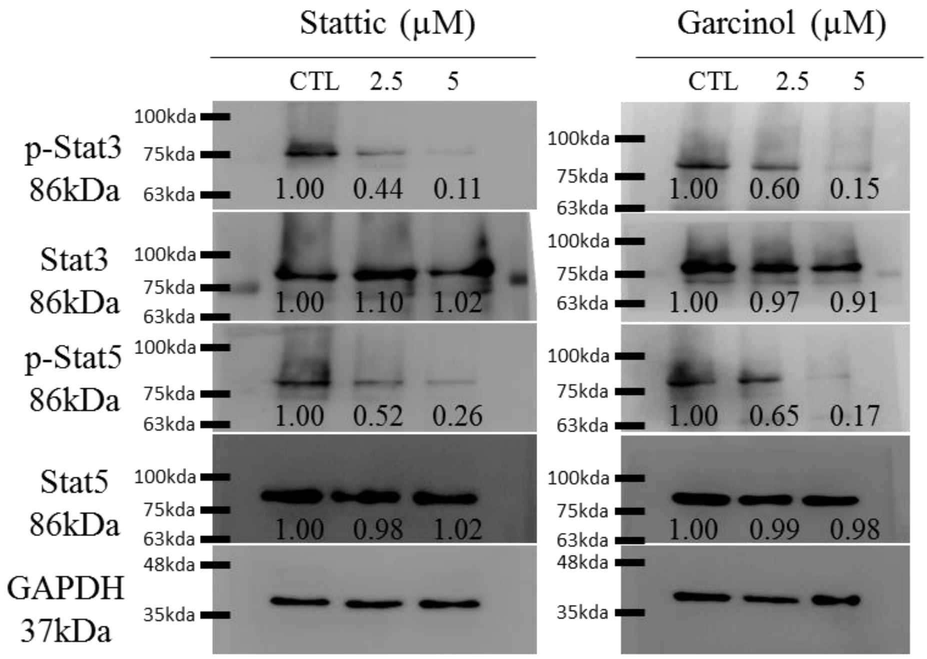

Figure S6. Full-size blots of Figure 6B.

© 2019 by the authors. Licensee MDPI, Basel, Switzerland. This article is an open access article distributed under the terms and conditions of the Creative Commons Attribution (CC BY) license (http://creativecommons.org/licenses/by/4.0/).
